# Optimal Method for Fetal Brain Age Prediction Using Multiplanar Slices From Structural Magnetic Resonance Imaging

**DOI:** 10.3389/fnins.2021.714252

**Published:** 2021-10-11

**Authors:** Jinwoo Hong, Hyuk Jin Yun, Gilsoon Park, Seonggyu Kim, Yangming Ou, Lana Vasung, Caitlin K. Rollins, Cynthia M. Ortinau, Emiko Takeoka, Shizuko Akiyama, Tomo Tarui, Judy A. Estroff, Patricia Ellen Grant, Jong-Min Lee, Kiho Im

**Affiliations:** ^1^Department of Electronic Engineering, Hanyang University, Seoul, South Korea; ^2^Fetal Neonatal Neuroimaging and Developmental Science Center, Boston Children’s Hospital and Harvard Medical School, Boston, MA, United States; ^3^Division of Newborn Medicine, Boston Children’s Hospital and Harvard Medical School, Boston, MA, United States; ^4^USC Mark and Mary Stevens Neuroimaging and Informatics Institute, University of Southern California, Los Angeles, CA, United States; ^5^Department of Radiology, Boston Children’s Hospital and Harvard Medical School, Boston, MA, United States; ^6^Computational Health Informatics Program, Boston Children’s Hospital and Harvard Medical School, Boston, MA, United States; ^7^Department of Neurology, Boston Children’s Hospital and Harvard Medical School, Boston, MA, United States; ^8^Department of Pediatrics, Washington University in St. Louis, St. Louis, MO, United States; ^9^Mother Infant Research Institute, Tufts Medical Center, Boston, MA, United States; ^10^Center for Perinatal and Neonatal Medicine, Tohoku University Hospital, Sendai, Japan; ^11^Department of Biomedical Engineering, Hanyang University, Seoul, South Korea

**Keywords:** deep learning, fetal MRI, fetal brain, brain age, age prediction

## Abstract

The accurate prediction of fetal brain age using magnetic resonance imaging (MRI) may contribute to the identification of brain abnormalities and the risk of adverse developmental outcomes. This study aimed to propose a method for predicting fetal brain age using MRIs from 220 healthy fetuses between 15.9 and 38.7 weeks of gestational age (GA). We built a 2D single-channel convolutional neural network (CNN) with multiplanar MRI slices in different orthogonal planes without correction for interslice motion. In each fetus, multiple age predictions from different slices were generated, and the brain age was obtained using the mode that determined the most frequent value among the multiple predictions from the 2D single-channel CNN. We obtained a mean absolute error (MAE) of 0.125 weeks (0.875 days) between the GA and brain age across the fetuses. The use of multiplanar slices achieved significantly lower prediction error and its variance than the use of a single slice and a single MRI stack. Our 2D single-channel CNN with multiplanar slices yielded a significantly lower stack-wise MAE (0.304 weeks) than the 2D multi-channel (MAE = 0.979, *p* < 0.001) and 3D (MAE = 1.114, *p* < 0.001) CNNs. The saliency maps from our method indicated that the anatomical information describing the cortex and ventricles was the primary contributor to brain age prediction. With the application of the proposed method to external MRIs from 21 healthy fetuses, we obtained an MAE of 0.508 weeks. Based on the external MRIs, we found that the stack-wise MAE of the 2D single-channel CNN (0.743 weeks) was significantly lower than those of the 2D multi-channel (1.466 weeks, *p* < 0.001) and 3D (1.241 weeks, *p* < 0.001) CNNs. These results demonstrate that our method with multiplanar slices accurately predicts fetal brain age without the need for increased dimensionality or complex MRI preprocessing steps.

## Introduction

The human brain exhibits lifelong age-related changes under complex genetic and environmental factors (Rakic, 2004; Kremen et al., 2010; Rando and Chang, 2012; Alexander-Bloch et al., 2020). As age-related brain changes are region-specific (Raz et al., 2005; Yun et al., 2020) and are known to be related to cognitive and behavioral performances (Olness, 2003; Gale et al., 2004; Rogne et al., 2015), prediction of brain age using magnetic resonance imaging (MRI) may be a crucial biomarker for brain health. Prior studies using structural MRIs have successfully predicted brain ages in healthy adults and children (Franke et al., 2012; Cole and Franke, 2017; Aycheh et al., 2018; Madan and Kensinger, 2018). The brain ages were predicted in patients with Alzheimer’s disease (Franke and Gaser, 2012; Gaser et al., 2013), schizophrenia (Koutsouleris et al., 2014; Schnack et al., 2016), epilepsy (Pardoe et al., 2017), Down syndrome (Cole et al., 2017a), traumatic brain injury (Cole et al., 2015), multiple sclerosis (Cole et al., 2020), and preterm birth (Franke et al., 2012). These studies established age prediction methods for healthy brain development or senescence from children to adults and examined individual discrepancies between the predicted and chronological ages [predicted age difference (PAD); chronological age – predicted brain age]. Significantly larger PADs were found in patients, which is considered a risk factor for altered maturation or aging of the brain, than healthy subjects. Several longitudinal studies have shown that large PADs are related to disease severity, cognitive function decline, and future conversion to Alzheimer’s disease (Franke and Gaser, 2012; Gaser et al., 2013; Cole et al., 2020). Therefore, the accurate prediction of brain age can provide clinically relevant information on brain health to predict future risks and detect structural abnormalities associated with brain disorders.

The human fetal brain shows dynamic structural changes with gestational age (GA). Under tight genetic control, quantitative structural brain features, including brain volume, cortical gyrification, surface area, curvature, and sulcal depth, were strongly correlated with GA and small intersubject variations in typically developing (TD) fetuses (Rajagopalan et al., 2011; Clouchoux et al., 2013, 2012; Hu et al., 2013; Wright et al., 2014; Namburete et al., 2015; Wu et al., 2015; Lefèvre et al., 2016; Andescavage et al., 2017). Moreover, atypical structural features have been more frequently found in fetuses with ventriculomegaly, congenital heart disease, and Down syndrome than in TD fetuses (Clouchoux et al., 2012; Scott et al., 2013; Kyriakopoulou et al., 2014; Lefèvre et al., 2016; Jaimes et al., 2020; Tarui et al., 2020; Rollins et al., 2021; Yun et al., 2021). Therefore, prediction of fetal brain age based on structural MRI features may be useful for detecting abnormalities in brain development and the risk of adverse developmental outcomes. Previous studies have predicted fetal brain age using linear or non-linear regression models based on quantitative volumetric and surface features (curvature and sulcal depth) and obtained mean absolute errors (MAEs, mean absolute PADs) of 0.87 (Namburete et al., 2015), 0.43 (Wu et al., 2015), and 0.47 (Wright et al., 2014) weeks of chronological GA. However, complex MRI processing and manual intervention are required to extract quantitative fetal brain features. Because of the motion between slices, even in fast single-shot T2-weighted imaging, multiple sets of fetal MRI stacks of thick 2D slices in different orthogonal planes are usually acquired to obtain better diagnostic performance; further, retrospective interslice motion correction using slice-to-volume registration techniques has been performed for quantitative fetal brain analysis (Gholipour et al., 2010; Kim et al., 2010; Kuklisova-Murgasova et al., 2012; Rousseau et al., 2013; Uus et al., 2020). Furthermore, accurate brain tissue segmentation is necessary for many volume- and surface-based analyses, which often require manual interventions.

Deep learning models have been used over the past few years for brain age prediction without complex MRI processing or manual intervention (Cole et al., 2017b; Huang et al., 2017; Bermudez et al., 2019; Jonsson et al., 2019; Wang J. et al., 2019). A recent study proposed a fetal brain age prediction method (Shi et al., 2020) and tested several inputs for an attention-based deep ensemble model including 3D MRI, multiple transverse slices (using multi-channel array), and a single transverse slice (center slice of the brain). The fully 3D approach using MRIs showed an MAE of 0.998 weeks. The multi-channel approach, which was trained using seven different channels for different slices (three slices superior/inferior to the center slice) in section “2D Network Architecture,” yielded an MAE of 0.974 weeks. The single-slice model showed a lower MAE (0.767 weeks) than the 3D and multi-channel models (Shi et al., 2020). As mentioned above, interslice motion in fetal MRIs may cause noisy information of brain structures and disrupt precise brain age prediction when using 3D and multi-channel approaches. Another reason for the low prediction performance of 3D and multi-channel approaches may be related to an increase in the dimensionality of the data. Although the third dimension allows the use of more information, it requires more parameters and a larger sample size to obtain robust prediction performance (Fu et al., 2019; Bashyam et al., 2020). Thus, 3D and multi-channel approaches have limitations in accurately predicting brain age using fetal MRIs. However, using a 2D single transverse slice per fetus may also have some limitations in accurately predicting brain age owing to the lack of sufficient brain anatomical information and the risk of selecting a bad slice with motion artifacts, non-uniform signal, or low signal to noise.

In this study, we aimed to propose a new method for fetal brain age prediction in an attempt to overcome the limitations of previous approaches. Our method employs a 2D single-channel convolutional neural network (CNN) with multiplanar slices that utilizes 3D brain structural information and reduces the risk of error without interslice motion or an increase in dimensionality. Multiplanar slices from MRI stacks in different orthogonal directions were used as inputs for our CNN, and multiple age prediction results were generated for each fetus. For the aggregation of the multiple predictions, we used three measures of central tendency (mean, median, and mode) and compared their age prediction performances to determine the most appropriate measure. To validate the proposed method, we performed several experiments. First, we statistically evaluated the advantage of using multiplanar slices in age prediction and compared it to that of using a single slice and a single stack. Second, we tested the effects of different inputs on age prediction accuracy. Multi-channel and 3D approaches were implemented, and their prediction performances were compared with those of our 2D single-channel CNN with multiplanar slices. Third, we generated saliency maps in individual brains to identify regions that significantly contribute to fetal brain age prediction. Lastly, we performed the same experiments on an external MRI dataset acquired from a different site and magnetic field strength.

## Materials and Methods

### Subjects and Image Acquisition

The use of fetal MRIs was approved by the institutional review board of Boston Children’s Hospital (BCH). Fetuses were collected by (1) prior prospective recruitment studies at BCH and (2) clinical fetal MRIs that were obtained to screen for fetal brain abnormalities but were clinically interpreted as normal by two board-certified radiologists experienced in fetal MRI. TD fetuses with maternal age between 18 and 45 years were included in this study. The exclusion criteria were multiple gestation pregnancies, dysmorphic features on ultrasound (US) examination, brain malformations/lesions or other identified organ anomalies on US examination, known chromosomal abnormalities, known congenital infections, and any clinically significant abnormality on visual inspection of the fetal MRI. After excluding 45 fetuses according to the exclusion criteria, we finally included 220 TD fetuses {GA: 29.1 ± 5.23 weeks [mean ± standard deviation (SD)], range: 15.9–38.7 weeks; sex: 109/86/25 (male/female/unknown)}.

Gestational age was estimated on the basis of the recommendations from American College of Obstetricians and Gynecologists and Committee on Obstetric Practice (2017). In the first trimester (<14 weeks of gestation), the crown rump length (CRL) and first day of the last menstrual period (LMP) were used. If they disagreed or LMP was uncertain, only CRL was used. Other US biometry measures, such as biparietal diameter, head circumference, abdominal circumference, and femur length, were used for those without estimated GAs in the first trimester.

Fetal brain MRIs were acquired on a Siemens 3T Skyra scanner using a T2-weighted half-fourier acquisition single-shot turbo spin-echo (HASTE) sequence with an in-plane resolution of 0.8–1.5 mm, a field of view (FOV) of 256 × 204, 256 × 256, or 320 × 320, a repetition time of 1.4–2.0 s, an echo time of 100–120 ms, and slice thickness of 2–4 mm depending on fetal size, fetal motion, and signal to noise. In each fetus, the MRI stacks were acquired multiple times in different orthogonal planes (usually 9–15 times); thus, a total of 2,765 multiplanar MRI stacks from 220 fetuses were included in this study. The number of fetuses in each GA is shown in Supplementary Figure 1.

To test the performance of the method on unseen data, we also included an external fetal MRI dataset from Tufts Medical Center (TMC). The use of the external dataset was approved by the institutional review board of TMC. Fetal MRIs were screened by a board-certified radiologist and a child neurologist with fetal neurology training at the obstetric clinic at TMC. The inclusion/exclusion criteria for the TD fetuses from TMC were the same as those from BCH. We included 21 TD fetuses [GA: 25.8 ± 4.97 weeks (mean ± SD), range: 19.7–33.3 weeks; sex: 9/10/2 (male/female/unknown)] acquired on a Phillips 1.5T scanner using a T2-weighted single-shot turbo spin-echo (SSTSE) sequence with an in-plane resolution of 1 mm, an FOV of 256 mm, a repetition time of 12.5 s, an echo time of 180 ms, and a slice thickness of 2–4 mm. After localizing the fetal brain, we acquired 4–10 SSTSE scans in three orthogonal orientations.

### Magnetic Resonance Imaging Processing

Non-brain tissues were removed from the fetal MRIs using our in-house tool based on 2D U-Net deep learning architecture.^1^ A total of 15,682 2D multiplanar slices from 397 MRIs with manually drawn masks [81 TD fetuses (28.0 ± 4.30 weeks of GA): a subset of our 220 fetuses for brain age prediction] were used for training the 2D U-Net architecture for automatic brain masking (Ronneberger et al., 2015). The average Dice coefficient between the manual and automatic brain masks was 0.9328, indicating the high accuracy of our in-house automatic brain masking tool. After brain extraction, we cropped the fetal MRI and had 12–60 slices in each cropped MRI stack. To correct intensity inhomogeneity, we applied N4 bias field correction to the masked brain region (Tustison et al., 2010). Thereafter, the noise was removed using a non-local mean denoising algorithm (Coupé et al., 2006), and the in-plane voxel size was resampled to 1 × 1 mm^2^. Finally, the resampled slices were unified using zero padding for use as an input to a deep learning network.

### 2D Network Architecture

**FIGURE 1:**
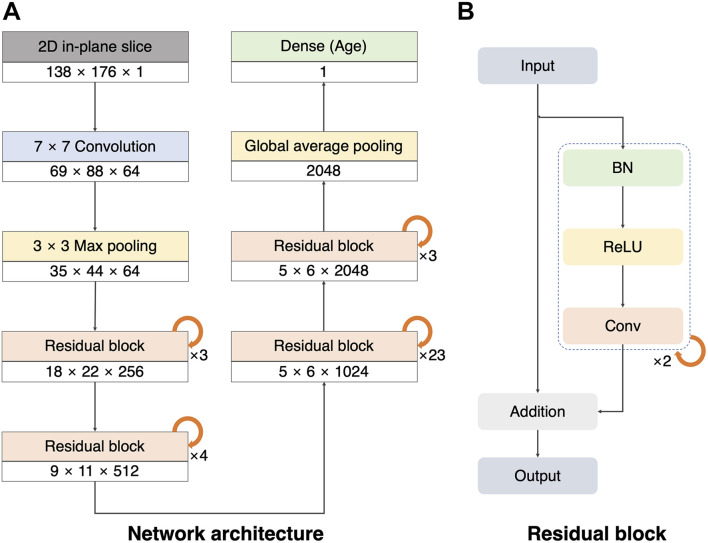
Architecture of our 2D single-channel convolutional neural network (CNN). **(A)** Architecture of the ResNet101V2 model. The size and number of the feature maps used in each step are listed at the bottom of the block (width × height × number of feature maps). Global average pooling compresses the final feature maps to a 2048 one-dimensional array. The dense layer was used to make a single regression output (brain age). **(B)** Residual block with batch normalization (BN), rectified linear unit (ReLU), and convolution (Conv).

To build a 2D single-channel CNN for brain age prediction, we modified ResNet101V2 (He et al., 2016; Figure 1A). We used the original version of the ResNet101V2 model as the layers before the last pooling. We replaced the last pooling of ResNet101V2 with the global average pooling to reduce model complexity and overfitting owing to the large number of layers used in our network model. Thereafter, a dropout layer (rate = 0.3) was created to prevent overfitting (Srivastava et al., 2014), and a dense layer was added to obtain the final prediction value. The convolutional layers created feature maps that reduced the size of the 2D slice from 138 × 176 to 5 × 6. The global average pooling and dense layer reduced the feature maps to age regression. The residual block comprised a combination of batch normalization, rectified linear unit (ReLU), and convolution (Figure 1B; Nair and Hinton, 2010; Ioffe and Szegedy, 2015). This pre-activation unit had a regularization effect because the inputs to all weight layers were normalized (He et al., 2016). The implementation details and hyperparameters were the same as those of the original ResNet101V2 (He et al., 2016). The batch size was set to 80, and the weights were initialized using a robust method proposed in a previous study (He et al., 2015), and Adam (learning rate = 0.1, decay = 0.001) was used as the optimizer (Kingma and Ba, 2015).

### Test-Time Augmentation

**FIGURE 2:**
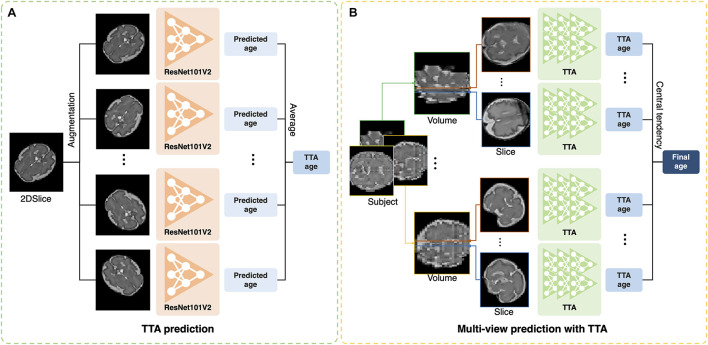
Illustration of test-time augmentation (TTA) and brain age prediction using multiplanar slices. **(A)** TTA creates multiple predictions by augmentation of a single slice and averages them to improve accuracy. **(B)** Multiplanar slices in orthogonal directions are used to predict brain ages. The measures of central tendency for multiple predictions from the multiplanar slices were calculated after TTA.


The test-time augmentation (TTA) technique was adopted to reduce the prediction error by combining multiple predictions generated by augmentation (Matsunaga et al., 2017; Jin et al., 2018). Using TTA, we created and averaged multiple predicted brain ages in each slice (Figure 2A); the formula is described as follows:

$$p_{s,v}^{TTA}=\frac{1}{N_{TTA}}\sum_{t}^{N_{TTA}}p_{s,v,t}$$

where *p*_s,v,t_ is the predicted age in *s*-th slice from the *v*-th MRI stack in the *t*-th augmentation, and $p_{s,v}^{\text{TTA}}$ is the average of multiple brain ages from the total number of augmentation (*N*_TTA_). Augmentation included the vertical flip, horizontal flip, width shift range (0.2), height shift range (0.2), rotation range (0–360), and intensity shift (0.5–1.0). TTA can eliminate the effects of arbitrary brain orientation, position, and random errors from a single prediction. To determine the optimal number of TTAs for brain age prediction, we obtained the MAE of each slice by changing the number of TTAs (1, 5, 10, 15, 20, 25, and 30). The MAE was expected to decrease as the number of TTAs increased until a specific number was reached. We found the specific number and used it as the optimal number of TTAs in this study.

### Central Tendency for Multiple Age Predictions

Previous deep learning studies have shown that the aggregation of the results from multiple predictions of 3D MRIs improved segmentation accuracy (Roy et al., 2019; Hong et al., 2020). In our study, the multiplanar slices had their own brain ages predicted by our 2D single-channel CNN after TTA (Figure 2B). We tested various strategies for aggregation of multiple brain age predictions. We obtained the brain age using the mean value of multiple predictions. We also calculated the median value of the multiple predictions to eliminate outliers among the predicted brain ages from noisy slices. Furthermore, we used a mode that aims to identify the value that occurs most frequently in a series. For the continuous variables, the mode of the predicted brain ages (*M*) was calculated using the following equation (Pearson, 1895):


$$M=\;L+i\cdot \left(f_{1}-f_{0}\right)/\left(2\cdot f_{1}-f_{0}-f_{2}\right)$$

where *L* represents the lower limit of the modal class (most frequent class); *f*_1_ is the frequency of the modal class; *f*_0_ is the frequency of the pre-model class; *f*_2_ is the post-modal class; and *i* is the class interval. In situations with two or more modal classes, the mode is calculated by three medians – two means of the predicted brain ages.

### Training Strategy

Our study was conducted using 2,765 MRI stacks obtained from 220 fetuses *via* 10-fold cross-validation. For our 2D single-channel CNN with multiplanar slices, we selected four nearest slices from the center of the brain in each stack. Therefore, 11,060 slices were used. In each training session, we selected 10% of our fetuses and their slices as the test dataset and used the rest of the fetuses/slices as the training dataset for our network model. In the training dataset, 10% of the fetuses and their slices were used as the validation dataset. The stratified sampling method was used to match the GA distribution between the training and test datasets, and oversampling was applied to the training and validation datasets for a balanced distribution of GA. To increase the number of training data, we applied a data augmentation technique using the same strategy described in section “Test-Time Augmentation.” Huber loss (*L*) was used as the loss function because it is less sensitive to outliers in the data (Huber, 1964). It selects the MAE or mean squared error (MSE) by a parameter (δ) of the absolute difference between the ground truth (*y*) and predicted value [*f*(*x*)] to reduce sensitivity and to determine the minima effectively:


$$L(y,f\left(x\right))=\;\left\{ \begin{array}{l} \;\;\;\frac{1}{2}{\left(y-f\left(x\right)\right)}^{2},\;\;\;if\;\left|y-f\left(x\right)\right|<\delta \\ \delta \left|y-f(x)\right|-\frac{1}{2}\delta^{2},\;\;\;\text{otherwise}\;\;\;\;\; \end{array}\right.$$

In this study, we selected a δ of 1.0. Optimal network weights on each fold were set by an early stopping and checkpoint callback function that monitored the MAE of the validation datasets across all epochs until it no longer improved over the last 150 epochs.

### Implementation of 2D Multi-Channel and 3D Deep Learning Approaches

**FIGURE 3:**
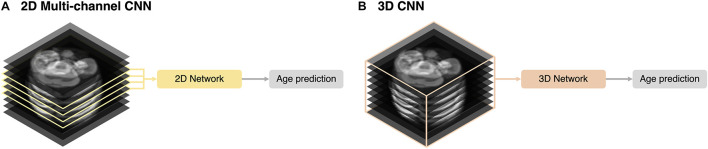
Schematic representation of the input strategies. **(A)** 2D multi-channel. **(B)** 3D volume approaches.

We implemented 2D multi-channel and 3D networks for different inputs for brain age prediction. Using the same network described in section “2D Network Architecture,” we trained multiple channels (multi-channel approach) with four slices selected by the same criteria used for the 2D single-channel network (Figure 3A). A 3D ResNet was employed to train a 3D network using the entire stack (3D approach) (Figure 3B). For the 3D network architecture, we adopted the same weights and depth as in the 2D CNN (Figure 1) and changed the 2D convolutional layers to 3D. However, owing to limited graphics processing unit (GPU) memory, we reduced the number of batches during the training from 80 (2D network) to 9. For both 2D multi-channel and 3D approaches, we used the same MRI preprocessing steps, TTA, and training strategy, including loss function and data augmentation described in the sections above. Similar to 2D data augmentation, only in-plane rotation was performed to minimize interpolation and resampling artifacts from the large slice thickness of the fetal MRI.

### Statistical Analyses

To evaluate the accuracy of the brain age predictions using different measures of central tendency (mean, median, and mode), we calculated the PAD of each measure by subtracting the predicted brain age from the chronological GA, and the MAE across the fetuses was computed. For the mode, we employed 10 different class intervals (0.1–1.0 week) and selected the one with the lowest MAE. We then compared the absolute PADs among the measures of central tendency using the Friedman test (Friedman, 1937) and *post hoc* Wilcoxon signed-rank test (Wilcoxon, 1945). We obtained the *R*^2^ value of the linear regression between the predicted brain age and chronological GA for the most accurate measure. To determine the difference in the prediction accuracy across GAs, we classified the fetuses into nine groups based on their chronological GAs. Fetuses under 23 weeks of gestation were grouped together, and the remaining fetuses were grouped in 2-week periods. A total of nine age groups were generated, and their absolute PADs were compared using the Kruskal–Wallis test (Kruskal and Wallis, 1952).

The effects of using multiplanar slices on brain age prediction were evaluated by comparing the distributions of the mean and SD of the absolute PADs of a single slice and a single stack. In each fetus, a single slice and a single stack were randomly selected, and their brain ages were obtained. For a single stack, the brain age was calculated by averaging the predictions of the four nearest slices from the center of the brain. Using the predicted brain ages from a single slice and a single stack, we calculated the MAEs by averaging the absolute PADs across the subjects and generated the distribution of the MAEs after 10,000 times of random selections. The SD of the absolute PADs across the subjects and its distribution were also examined to evaluate the variance of errors. We counted the number of test statistics with lower MAEs or SDs other than those from our method and calculated the *p*-value of the random selection tests using the proportion of the number of cases out of 10,000.

We statistically compared the performance in brain age prediction among the different input strategies in the deep learning networks (2D single-channel, 2D multi-channel, and 3D approaches). The input of the 3D approach was the entire MRI stack and generated the predicted brain age in a stack. The 2D multi-channel approach treated four slices from a stack as the channels and generated a predicted brain age in a stack. Unlike the 2D multi-channel and 3D approaches, the 2D single-channel CNN generated a predicted brain age in each of the multiple slices. Thus, to correctly compare the prediction performance among these approaches, we averaged four predictions using our single-channel CNN to obtain the predicted brain age in each stack. We then measured the absolute PADs for each stack of all subjects and compared them between our single-channel CNN and other approaches using the Wilcoxon signed-rank test (Wilcoxon, 1945).

The brain ages of the external TD fetuses were predicted using our 2D single-channel CNN. Using different aggregation strategies, we obtained the MAEs and *R*^2^ values of linear regression between the predicted brain age and GA from the external TD fetuses. From the external dataset, the distributions of the mean and SD of the absolute PADs of a single slice and a single stack after 10,000 random selections were generated to evaluate the effects of using multiplanar slices. We also compared the absolute PADs of our single-channel CNN to those of the 2D multi-channel and 3D approaches using the Wilcoxon signed-rank test.

### Visualization of the Age Prediction Model and Evaluation of the Brain Size Effect

The saliency visualization method was employed to approximate the brain regions that significantly influence the prediction of brain age (Simonyan et al., 2013; Oh et al., 2019). The saliency in this study was calculated using backpropagation to track the brain regions connected to large weights. To obtain the explainable visualization map, we applied Gaussian smoothing [sigma = (1.5, 1.5), order = 0] and normalization using maximum saliency values. In this study, saliency maps were generated in each input slice, and visual inspection of the maps was performed to identify the common regions with high saliency across all slices.

In addition to saliency maps, we examined the effect of brain size on age prediction, since fetal brain volume is highly correlated with GA (Huang et al., 2009; Clouchoux et al., 2012). In each fold, we reduced the brain size in each slice of the test dataset with five isometric scaling from 0.5 to 0.9 and then predicted the brain ages with the reduced brain size. Linear regression analyses were performed between the brain age using the reduced brain size and GA.

## Results

### Fetal Brain Age Prediction With Multiplanar Slices

Supplementary Figure 2 shows the experimental results of the effects of the number of TTAs. As the number increased, the MAE decreased until 20 and showed no change thereafter. Thus, we selected “20 times the TTA” for brain age prediction.

**FIGURE 4:**
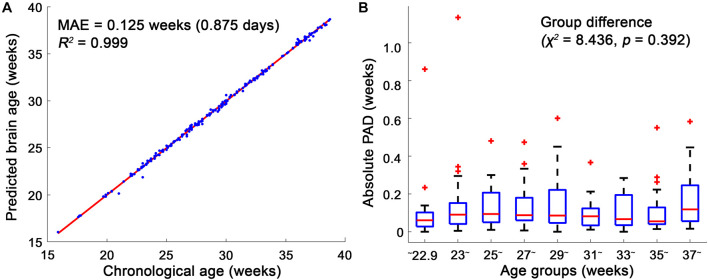
Linear regression model between the chronological age and brain age and box plots of the absolute predicted age differences (PADs) in the age groups. **(A)** The mean absolute error (MAE) between the chronological and brain ages was 0.125, and their regression coefficient (*R*^2^) was 0.999. **(B)** Among the age groups, no significant difference in the absolute PADs was found in the Kruskal–Wallis test. In each age group, the red horizontal line indicates the median of the absolute PADs, and the bottom and top edges of the box represent the lower quartile and upper quartile of the absolute PADs. The outliers (red crosses) represent the values that fall outside of the lower or upper boundaries between 1.5 times of the interquartile range, respectively. The black horizontal lines display the boundaries.

Among the 10 different class intervals, the mode with 0.2 weeks of class interval showed the lowest MAE [0.125 weeks (0.875 days); Table 1]. The MAEs increased with class intervals from 0.4 to 1.0, while similar MAEs were found with class intervals between 0.1 and 0.4 weeks. The mean and median showed MAEs of 0.236 and 0.152 weeks, respectively. The Friedman test showed significantly different absolute PADs among the three measures (χ^2^ = 88.26, *p* < 0.001), and the mode with 0.2 weeks of class interval had a significantly lower absolute PAD than the mean (*z* = −8.153, *p* < 0.001) and median (*z* = −3.211, *p* = 0.001). With the use of the mode with 0.2 weeks of class interval, the regression analysis between the predicted brain age and chronological GA showed an *R*^2^ value of 0.999 and *p*-value of <0.001 (Figure 4A). There was no significant difference in the absolute PADs among the different age groups (χ^2^ = 8.436, *p* = 0.392) in the Kruskal–Wallis test (Figure 4B).

**TABLE 1:** Prediction performances using different measures of central tendency for multiple age predictions.

Measure		MAE (weeks) ± SD

Mean		0.236 ± 0.246
Median		0.152 ± 0.162
**Mode**	**Class intervals (weeks)**	
	
	0.1	0.126 ± 0.148
	0.2	0.125 ± 0.141^†^
	0.3	0.130 ± 0.166
	0.4	0.142 ± 0.183
	0.5	0.144 ± 0.154
	0.6	0.162 ± 0.191
	0.7	0.179 ± 0.144
	0.8	0.191 ± 0.152
	0.9	0.207 ± 0.144
	1	0.220 ± 0.183

*^†^Lowest mean absolute error (MAE) ± standard deviation (SD). The mode with 0.2 weeks of class interval showed a significantly lower MAE than the mean and median.*

**FIGURE 5:**
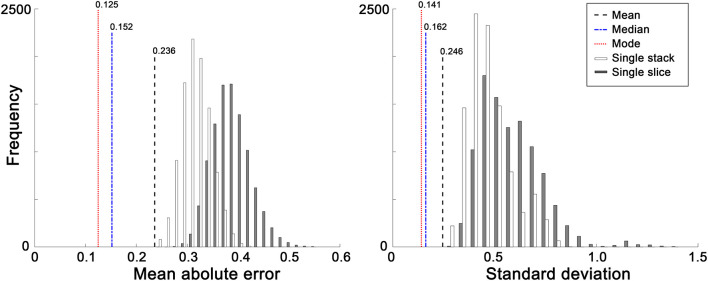
Distributions of the averages and standard deviations of the absolute predicted age difference (PAD) using a single volume or a single slice. During 10,000 random selections, a single volume and a single slice were randomly selected in each subject. The mean absolute errors (MAEs) and standard deviations obtained using our method (mean, median, and mode) were significantly lower than those using a single volume or a single slice (*p* < 0.001).

The distributions of the means and SDs of the absolute PADs calculated from the randomly selected slices and stacks are shown in Figure 5. The MAEs and SDs of our method with the three different measures (mean, median, and mode) were significantly lower than those of the single-slice and single-stack approaches (*p* < 0.001).

### Performance of the Deep Learning Networks With Other Inputs

To correctly compare the prediction performance among the different CNNs, we averaged the four predicted brain ages in each stack from our 2D single-channel CNN to obtain a stack-wise brain age as in the 2D multi-channel and 3D CNNs. Notably, the stack-wise MAE from our 2D single-channel CNN was lower than our final result (MAE of 0.125 weeks) because different aggregation strategies (mean) were applied, and a small number of slices for aggregation (four slices in each stack) were used for the stack-wise brain age. The stack-wise MAEs of our 2D single-channel method, 2D multi-channel, and 3D approaches were 0.304, 0.979, and 1.114 weeks, respectively (Table 2). For the stack-wise brain ages, the 2D single-channel method showed a significantly lower absolute PAD than the 2D multi-channel (*z* = −36.15, *p* < 0.001) and 3D (*z* = −45.10, *p* < 0.001) approaches.

**TABLE 2:** Prediction performances of the deep learning networks using different inputs.

Approaches	MAE (weeks) ± SD
2D single-channel*	0.304 ± 0.459^†^
2D multi-channel	0.979 ± 1.205
3D	1.114 ± 1.281

*The mean absolute error (MAE) and standard deviation (SD) of the 2D single-channel were obtained using the stack-wise brain ages. ^†^Significantly lower than that in the 2D multi-channel and 3D approaches.

### Brain Regional Influence and Whole Brain Size Effect on Age Prediction

**FIGURE 6:**
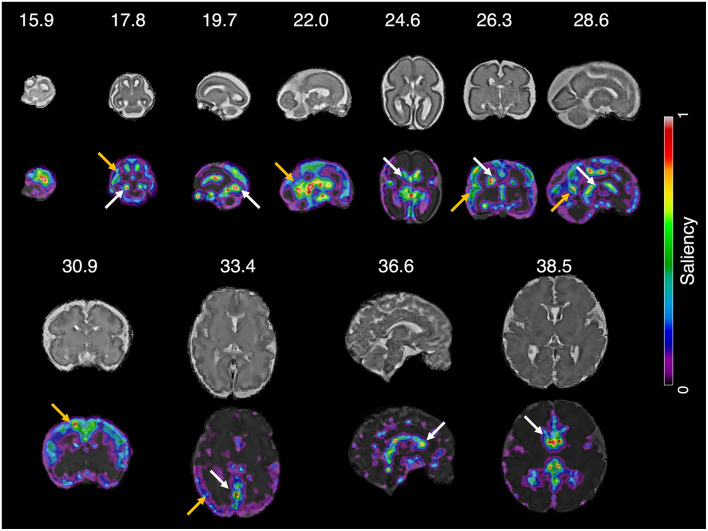
Saliency map of fetal brain age prediction. Large saliency represents important regions contributing to fetal brain age prediction. For all slices, the cortex (yellow arrows) and ventricles (white arrows) had larger saliency values than the other brain regions.

We determined which parts of the input slice were the primary contributors to predicting brain age using the saliency maps. Examples of the saliency maps are shown in Figure 6. After visual inspection of all the saliency maps, the ventricles had a high saliency value ranging from 0.5 (green) to 1.0 (red) in all slices. The cerebral cortex had a similar saliency value to the ventricles in the slices between 15.9 and 30.9 weeks of gestation. Although the cortex showed a smaller saliency value between 0.1 (purple) and 0.4 (light blue) in the last three slices over 33.5 weeks of gestation, it was still larger than the other brain regions. Therefore, we determined that the ventricles and cortex were important regions for fetal brain age prediction. Additionally, a strong relationship between brain size and brain age prediction was found (Supplementary Figure 3). The regression results showed that a greater reduction in brain size resulted in a smaller brain age.

### Performance on the External Data

Using the external fetal MRIs from TMC, we obtained an MAE of 0.508 weeks with the use of the mode with 0.2 weeks of class interval, which was lower than those with the use of the mean (MAE of 0.670 weeks) and median (MAE of 0.560 weeks). The mode also showed a high regression coefficient (*R*^2^ = 0.992) between the predicted brain age and GA (Supplementary Figure 4A). The MAE of our method using the mode was significantly lower than those of the randomly selected single slice (*p* = 0.005) and single stack (*p* = 0.007) approaches (Supplementary Figure 4B). A significantly lower SD was found with our method using the mode than the methods using the randomly selected single slice (*p* = 0.002) and single stack (*p* = 0.002) (Supplementary Figure 4C). Further, the stack-wise brain ages were obtained using our 2D single-channel, 2D multi-channel, and 3D CNNs, and their prediction performances were compared. Our 2D single-channel CNN showed a significantly lower stack-wise MAE (0.743 weeks) than the 2D multi-channel (1.466 weeks, *z* = −6.48, *p* < 0.001) and 3D (1.241 weeks, *z* = −5.67, *p* < 0.001) approaches.

## Discussion

In this study, we proposed a new method for predicting fetal brain age using structural fetal brain MRIs. To improve the prediction performance, we used multiplanar slices in different orthogonal directions and generated multiple brain age predictions from a single-channel CNN. For the aggregation of the multiple predictions from the multiplanar slices, we calculated various measures of central tendency, and the best performance was obtained with the mode with 0.2 weeks of class interval [MAE of 0.125 weeks (0.875 days)]. The proposed method with multiplanar slices showed a significantly lower MAE than those with a single slice and a single stack, and a significantly higher accuracy than the 2D multi-channel and 3D approaches. Furthermore, when applying our method to an external dataset acquired from a different site and field strength of MRI, we found significantly higher accuracy compared to a single slice, a single stack, 2D multi-channel CNN and 3D CNN approaches. Using the external test set from TMC, we found that the optimal method yielded an MAE of 0.506 weeks (3.556 days).

### High-Performance Fetal Brain Age Prediction Using the 2D Single-Channel Convolutional Neural Network With Multiplanar Slices

Previous studies have reported that the prediction of brain age has the potential to be a crucial biomarker for evaluating fetal development and health (Namburete et al., 2015; Wu et al., 2015). Although different datasets were used across the studies, the proposed method yielded a more accurate fetal brain age prediction (MAE = 0.125 weeks) than a deep learning network (MAE = 0.767 weeks) and regression models using the brain volume (MAE = 0.72 weeks), average curvedness (MAE = 0.43 weeks), and mean curvature *L*_2_ norm (MAE = 0.47 weeks) used in previous studies (Wright et al., 2014; Namburete et al., 2015; Wu et al., 2015; Shi et al., 2020). Our experimental results of the random selection tests showed that the multiplanar slices yielded a significantly lower mean and SD of the absolute PAD than a single slice and a single stack. This may be related to the utilization of 3D spatial information of the fetal brain that is not available in a single slice or stack. In addition to the 3D spatial information, the number of slices may be an important factor for obtaining a low prediction error and its variance. Signal intensity artifacts induced by unpredictable head motion and image degradation due to dielectric effects or poor receiver coil placement can be seen in some slices, which may produce outliers of brain age prediction when using a small number of slices. Using multiple slices may reduce the risk of highly deviated age predictions resulting from selecting a noisy MRI stack or slice by chance. To validate the performance of the proposed method, we evaluated the prediction accuracy of the external test set from TMC. We obtained an MAE of 0.508 weeks using our optimal method, which is lower than that in a previous study using a deep learning network (Shi et al., 2020).

Among the various measures of central tendency for multiple predictions, the mode with 0.2 weeks of class interval showed the lowest MAE compared with the mean and median. This suggests that identifying the most frequent class from multiple predictions can be an efficient aggregation strategy to exclude outliers from noisy slices. For the mode, because the large class intervals lose detailed patterns of the distribution, the MAE increases when using a large class interval (0.5 or larger). Conversely, small class intervals have the risk of generating irregular histograms with over-represented and empty classes. We found that 0.1 weeks of class interval showed a similar but higher MAE compared with 0.2 weeks. A class interval smaller than the minimum unit of GA [0.143 weeks (1 day)] may result in an irregular histogram and inappropriate mode estimation. We employed 0.2 weeks of class interval as the optimal size because it is the smallest one, which is larger than the minimum unit of GA and achieves the lowest MAE among the intervals.

Using our dataset, we implemented the 2D multi-channel and 3D CNNs used in a previous study (Shi et al., 2020), which may have an advantage in utilizing 3D spatial information. However, the 3D approach showed the lowest prediction accuracy among the three input methods. This may be related to an increase in the dimensionality of the data, which can leverage the interslice context but requires more parameters and sample size for reliable prediction performance (Fu et al., 2019; Bashyam et al., 2020). Compared to the in-plane resolution (0.8–1.5 mm), the slice thickness (2–4 mm) of fetal MRIs is relatively large for small fetal brains. Because of the large slice thickness, brain structures may not be recognized well, which may lower the performance of the 3D approach. Motion between slices in fetal MRIs may also produce incorrect spatial information, which affects the performance of the 3D volume approach. Similar to the 3D approach, the 2D multi-channel approach is sensitive to interslice motion artifacts because the feature map of a slice is calculated by the weighted summation of the feature maps of the adjacent slices using convolution. Thus, utilizing 3D spatial information without motion artifacts and an increase in dimensionality may allow us to obtain highly accurate brain age prediction results in our proposed 2D single-channel CNN with multiplanar slices.

### Overfitting Issue on the High Performance of Our Fetal Brain Age Prediction Method

Learning-based methods might exhibit an extremely high performance with overfitting of the training data. Because the proposed method was trained with single-site MRIs, our highly accurate brain age prediction (MAE = 0.125 weeks) could be a result of overfitting. However, we adopted several techniques in this study to increase the prediction performance without overfitting. First, we ensured that the trained network was robust to image contrast varying from different sites and magnetic field strengths. Because gray-scale intensity values from single-site MRIs show small variations, we increased the diversity of the image contrast of the data using intensity shift augmentation when training and testing the network. Second, several techniques were used to prevent overfitting in training our 2D single-channel CNN. In the network architecture, we adopted global average pooling, dropout, early stopping, and the pre-activation unit to minimize overfitting. Dropout, global average pooling, and early stopping are commonly used in deep learning studies using neuroimaging data (Zhang et al., 2015; Kamnitsas et al., 2017; Aycheh et al., 2018; Kushibar et al., 2018; Wachinger et al., 2018; Jonsson et al., 2019; Wang G. et al., 2019; Wang J. et al., 2019; Bashyam et al., 2020; Ebner et al., 2020; Hong et al., 2020; Shi et al., 2020). Furthermore, the regularization impact of the pre-activation unit was shown in a previous study (He et al., 2016). Using the abovementioned techniques, our 2D single-channel CNN yielded an MAE of 0.410 weeks when predicting brain age in a single slice (Supplementary Figure 2), which is similar to other studies on fetal brain age prediction (Wright et al., 2014; Wu et al., 2015). Our method showed a relatively lower MAE than a previous single-slice deep learning method (MAE = 0.767 weeks) (Shi et al., 2020). Generally, deep learning networks require a large amount of training data, and the amount of training data is correlated with the performance of deep learning networks (Zhu et al., 2016; Sun et al., 2017). Since we used a total of 11,060 multiplanar slices that is 15 times or more than (Shi et al., 2020), we assumed that a large number of slices for training may improve the prediction accuracy. Finally, after training, the prediction accuracy is further improved by postprocessing. Postprocessing is known to improve the results of learning-based methods (Liu et al., 2019; Roy et al., 2019; Hong et al., 2020). In fetal MRIs, the quality of some slices is not sufficient to obtain an accurate brain age because of motion artifacts, non-uniform signals, or low signal to noise. Aggregation of multiple results is commonly employed to improve accuracy (Roy et al., 2019; Hong et al., 2020) and has the potential to reduce the risk of erroneous predictions from low-quality slices. In this study, we applied several strategies for the aggregation of the multiple predictions from the multiplanar slices and found an optimal strategy to significantly reduce the MAE from 0.410 to 0.125 weeks. To the best of our knowledge, the postprocessing step is not associated with overfitting in deep learning networks. The significant effect of aggregation on age prediction accuracy was also shown in the external TMC MRI dataset obtained from a different site and field strength (1.5T). The MAE across the external MRI slices was 0.852 weeks; however, our optimal approach significantly reduced the prediction error (MAE = 0.508 weeks). This result suggests that aggregation of multiple brain age predictions is an optimal method for fetal brain age prediction to improve prediction performance. Nevertheless, the external TMC MRI dataset showed a higher MAE than our BCH dataset. Since we trained our 2D single-channel CNN with single-site MRIs, the model still has an overfitting issue for one particular MRI platform. Including a large sample of multi-site MRI datasets for training the CNN has the potential to improve the generalizability of the method.

### High Impact of Cortical and Ventricular Structures and Brain Size on Fetal Brain Age Prediction

The saliency maps showed that the cortex and ventricles are important regions for predicting fetal brain age. The cerebral cortex becomes highly convoluted as folding increases during gestation, and brain ages can be predicted by quantitative folding features, such as gyrification index, surface area, and curvature (Clouchoux et al., 2012; Wright et al., 2014; Wu et al., 2015). Previous studies have demonstrated that the ventricles are associated with fetal brain development. Enlargement of the anterior horns of the lateral ventricle was found in the second trimester, and thinning of the anterior, inferior, and posterior horns of the lateral ventricles was reported during pregnancy (Huang et al., 2009). Furthermore, shrinkage of the ventricle volume was observed in the late second trimester (Huang et al., 2009). These previous reports support that our prediction method identifies and uses the biologically relevant anatomical regions known to be closely related to fetal brain age. Compared to the saliency maps of a previous study (Shi et al., 2020), our saliency maps highlighted similar regions, such as the cerebral cortex and ventricles. While their saliency maps (Shi et al., 2020) showed lower saliency values in the ventricles than in the cerebral cortex, our saliency maps showed relatively similar values between these regions. This pattern suggests that the ventricles similarly contribute to predicting fetal brain age compared to the cerebral cortex. As the ventricles are associated with fetal brain development (Huang et al., 2009), the relatively higher saliency values in the ventricles may be related to the higher prediction accuracy of the proposed method.

In addition to the cortical folding structure and ventricle volume, we found that the whole brain size plays an important role in predicting fetal brain age. These results are supported by previous reports indicating that brain volume increases as gestation progresses (Huang et al., 2009; Clouchoux et al., 2012) and is a crucial factor for fetal brain age prediction (Wu et al., 2015).

### Limitation and Future Works

The proposed method showed a highly accurate prediction performance for fetal brain age in TD fetuses; however, there are limitations that should be investigated and considered in future studies. Compared to the MAEs, the SDs were relatively large across all experiments. To determine the reason for the large SDs, we performed supplementary experiments to obtain the MAEs and SDs after excluding the upper 5% of the absolute PADs. The results indicated that large SDs may be associated with outliers (Supplementary Tables 1, 2). In the supplementary experiments, two fetuses consistently had an upper 5% of the absolute PADs without any specific reasons, such as brain malformation and low MRI quality. In our future study, we will investigate unrevealed factors affecting incorrect brain age prediction.

A large sample of multi-site MRI datasets for training a CNN has potential in improving the generalizability of the method. Demographic factors, such as parental race and ethnicity, are related to fetal growth (Bottomley and Bourne, 2009; Buck Louis et al., 2015) which may influence age prediction. Thus, their effects on fetal brain age prediction should be investigated using large datasets in future studies. In our dataset, a small number of TD fetuses under 21 weeks of GA were included. Although we employed stratified sampling to balance the GA distribution between the training and test datasets, the imbalanced GA distribution of our dataset may be associated with the prediction errors in our results. A dataset with a balanced GA distribution is necessary to improve the performance in brain age prediction.

The batch size of the 3D CNN (*n* = 9) was smaller than that of the 2D single-channel CNN (*n* = 80) because of hardware limitations. Small batches are thought to increase the prediction performance (Arpit et al., 2016; Radiuk, 2017; Masters and Luschi, 2018; Kandel and Castelli, 2020) but may have a risk of causing overfitting issue. To precisely assess the prediction performance of the 3D CNN, we need more hardware resources to apply the same batch size used in the 2D single-channel CNN to the 3D CNN. Moreover, the hyperparameters used in the 3D CNN were the same as those used in the 2D CNN. The prediction performance of a 3D CNN can be further improved by optimizing the architecture and hyperparameters (Payan and Montana, 2015).

Our proposed method should be applied to clinically abnormal fetuses with clinical interpretation to evaluate the clinical relevance of PADs. In future studies, the relationship between PADs and neurodevelopmental outcomes after birth should be investigated. Finally, systematic errors may occur in GA estimation with any LMP- or US biometry-based approaches (Ioannou et al., 2012; Oppenraaij et al., 2015). Since GA was used as the gold standard, the PAD might be affected by systematic errors in GA estimation.

## Data Availability Statement

Due to the institutional restrictions, the data in this study is only accessible with a data-sharing agreement. The main code used in this work is publicly available on Github repository (https://github.com/jwhong1125/Fetal_BrainAge). Further inquiries can be directed to the corresponding author/s.

## Ethics Statement

The use of fetal MRIs was approved by the Institutional Review Boards at the Boston Children’s Hospital (BCH) and Tufts Medical Center (TMC). Written informed consent to participate in this study was provided by the participants’ legal guardian/next of kin.

## Author Contributions

JH, HY, KI, and J-ML designed the main idea, directed the overall analysis, and wrote the manuscript with input from all authors. JH and HY developed the algorithm and performed the data processing and experiments. GP, SK, YO, LV, CR, CO, ET, SA, TT, JE, and PG assisted with the data collection and result interpretation. All authors contributed to the article and approved the submitted version.

## Conflict of Interest

The authors declare that the research was conducted in the absence of any commercial or financial relationships that could be construed as a potential conflict of interest.

## Publisher’s Note

All claims expressed in this article are solely those of the authors and do not necessarily represent those of their affiliated organizations, or those of the publisher, the editors and the reviewers. Any product that may be evaluated in this article, or claim that may be made by its manufacturer, is not guaranteed or endorsed by the publisher.

## Footnotes

1
https://github.com/FNNDSC/fetal-brain-segmentation
