# Supplementary material for: Optimal Method for Fetal Brain Age Prediction Using Multiplanar Slices From Structural Magnetic Resonance Imaging
Source: Front Neurosci. 2021 Oct 11;15:714252. doi: 10.3389/fnins.2021.714252 (PMC8542770; doi:10.3389/fnins.2021.714252)
Supplement: Supplementary file 1 [file Data_Sheet_1.docx]

[Supplementary materials]

Optimal Method for Fetal Brain Age Prediction
using Multiplanar Slices from Structural Magnetic Resonance Imaging

Jinwoo Hong^1,2†^, Hyuk Jin Yun^2,3†^, Gilsoon Park^4^, Seonggyu Kim^1^, Yangming Ou^2,3,5,6^, Lana Vasung^2,3^, Caitlin K. Rollins^7^, Cynthia M. Ortinau^8^, Emiko Takeoka^9^, Shizuko Akiyama^10^, Tomo Tarui^9^, Judy A. Estroff^5^, P. Ellen Grant^2,3,5^, Jong-Min Lee^11*^, and Kiho Im^2,3^

^1^ Department of Electronic Engineering, Hanyang University, Seoul 04763, South Korea

^2^ Fetal Neonatal Neuroimaging and Developmental Science Center, Boston Children’s Hospital, Harvard Medical School, Boston, MA 02115, USA

^3^ Division of Newborn Medicine, Boston Children’s Hospital, Harvard Medical School, Boston, MA 02115, USA

^4^ USC Mark and Mary Stevens Neuroimaging and Informatics Institute, University of Southern California, Los Angeles, CA 90033, USA

^5^ Department of Radiology, Boston Children’s Hospital, Harvard Medical School, Boston, MA 02115, USA

^6^ Computational Health Informatics Program, Boston Children’s Hospital, Harvard Medical School, Boston, MA 02115, USA

^7^ Department of Neurology, Boston Children’s Hospital, Harvard Medical School, Boston, MA 02115, USA

^8^ Department of Pediatrics, Washington University in St. Louis, St. Louis, MO 63110, USA

^9^ Mother Infant Research Institute, Tufts Medical Center, Boston, MA 02111, USA

^10^ Center for Perinatal and Neonatal Medicine, Tohoku University Hospital, Sendai 980-8574, Japan

^11^ Department of Biomedical Engineering, Hanyang University, Seoul 04763, South Korea

*** Corresponding authors**

**Jong-Min Lee, Ph.D**

Dept. of Biomedical Engineering, Hanyang University

Sanhakgisulkwan # 319, 222 Wangsimni-ro, Seongdong-gu, Seoul, 04763, South Korea

Tel.: +82-2-2220-0685

Email: ljm@hanyang.ac.kr

**† Co-first authors**

Jinwoo Hong and Hyuk Jin Yun contributed equally to this work.

**Keywords:** deep learning, fetal MRI, fetal brain, brain age, age prediction**Supplementary** **Table 1. Prediction performances using different measures of central tendency for multiple age predictions after excluding upper 5% of absolute predicted age difference**

| Measure | | MAE (weeks) ± SD |
| --- | --- | --- |
| Mean | | 0.197 ± 0.160 |
| Median | | 0.124 ± 0.101 |
| Mode | Class intervals (weeks) |  |
|  | 0.1 | 0.102 ± 0.094 |
|  | 0.2 | 0.102 ± 0.085 |
|  | 0.3 | 0.101 ± 0.085 |
|  | 0.4 | 0.108 ± 0.086 |
|  | 0.5 | 0.118 ± 0.090 |
|  | 0.6 | 0.129 ± 0.102 |
|  | 0.7 | 0.158 ± 0.108 |
|  | 0.8 | 0.169 ± 0.108 |
|  | 0.9 | 0.187 ± 0.108 |
|  | 1 | 0.190 ± 0.123 |

MAE: mean absolute error and SD: standard deviation.

**Supplementary Table 2. Prediction performances of deep learning networks using different inputs after excluding upper 5% of absolute predicted age difference**

| Approaches | MAE (weeks) ± SD |
| --- | --- |
| 2D single-channel | 0.228 ± 0.222 |
| 2D multi-channel | 0.787 ± 0.617 |
| 3D | 0.905 ± 0.699 |

MAE: mean absolute error and SD: standard deviation. The MAE and SD of 2D single-channel were obtained by the stack-wise brain ages.

**Supplementary Figure S1. The number of fetuses in each gestational age (GA).**


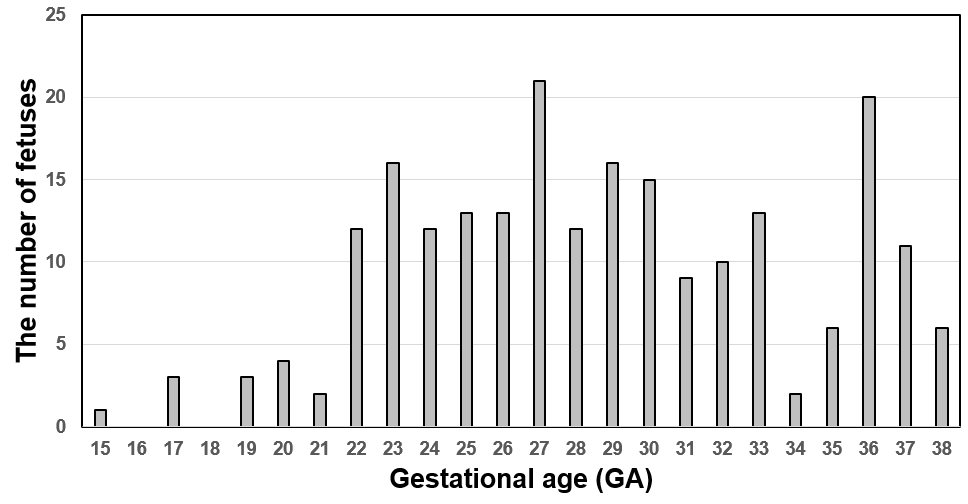


**Supplementary Figure S2. The mean absolute error (MAE) of brain age prediction by changing the number of test time augmentation (TTA).** The MAE decreased until 20 and showed no change after that (0.410 weeks).


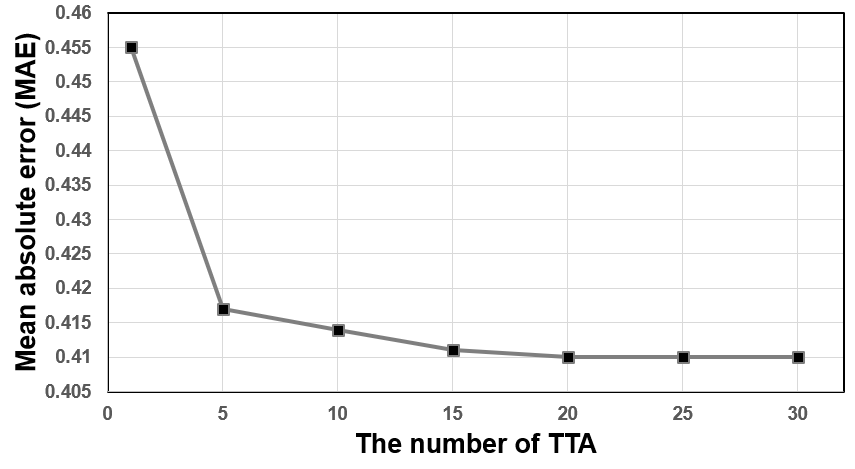


**Supplementary Figure S3. Relationship between brain size and age prediction.** In each slice, brain size was reduced with five isometric scaling from 0.5 to 0.9. Scaling of 1.0 is original brain size. Linear regression analyses were performed between predicted brain age using the reduced brain size and GA.


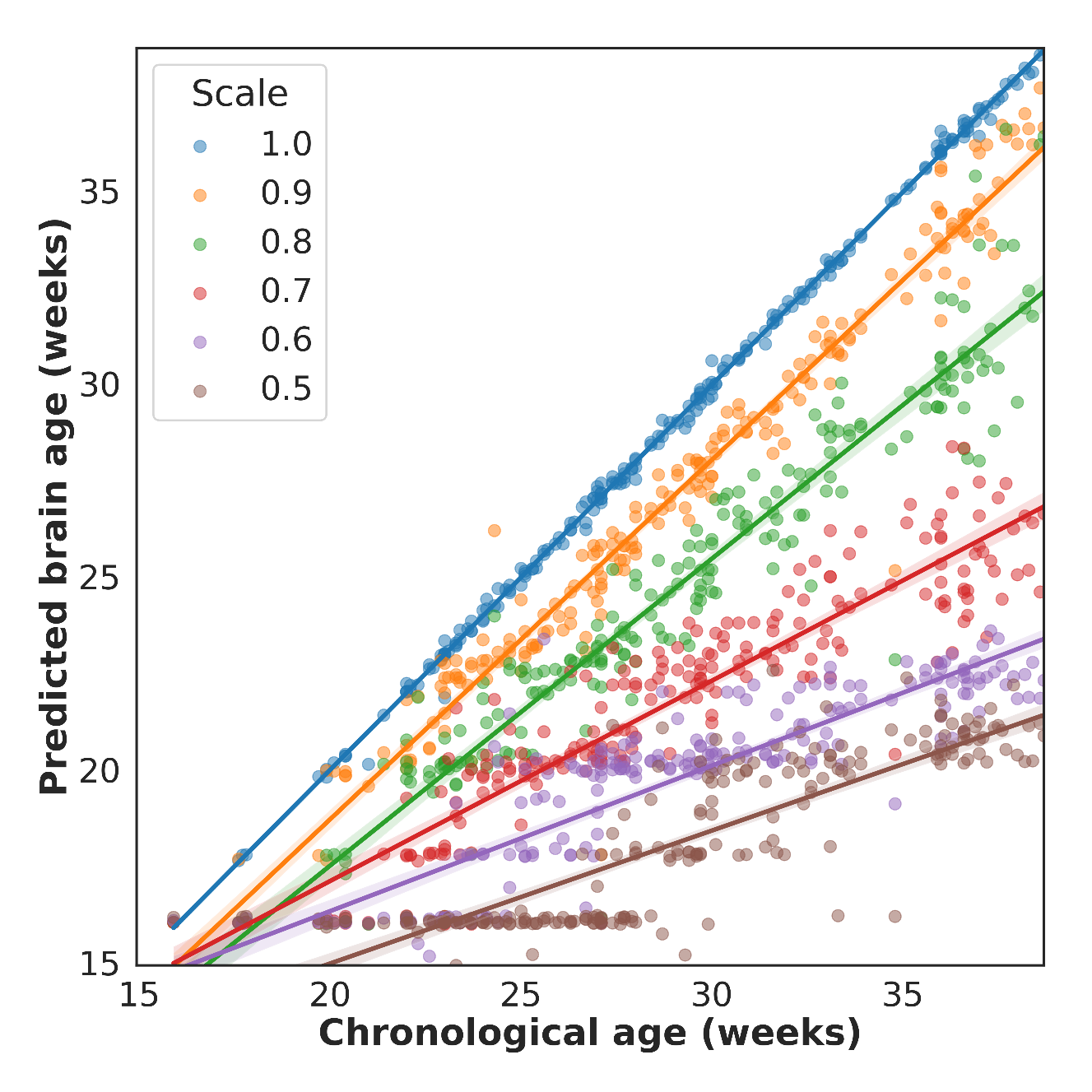


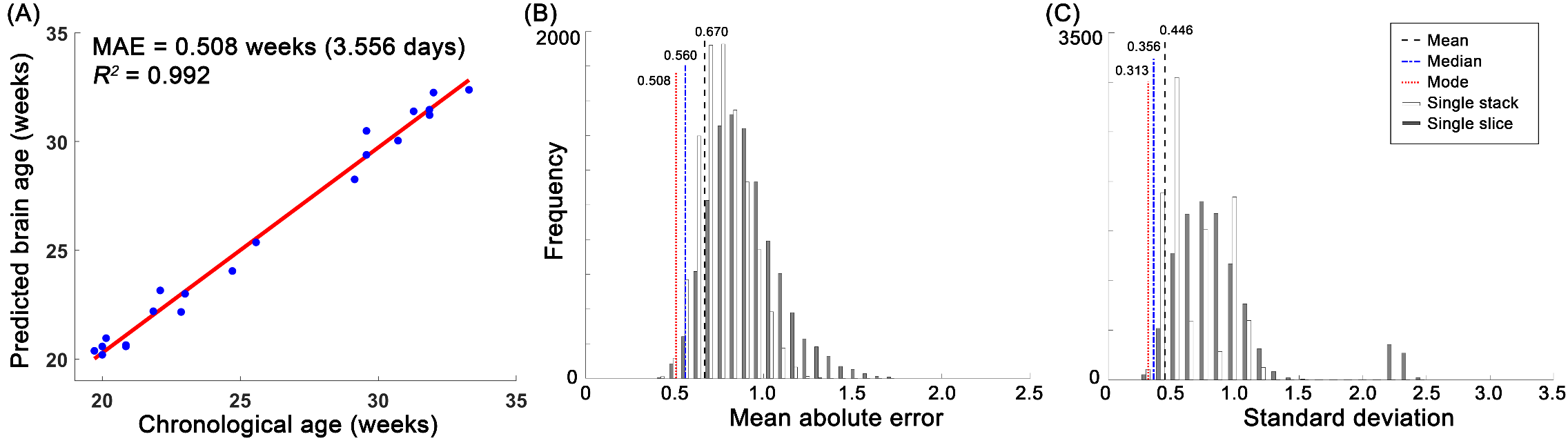
**Supplementary Figure S4. Results of performance on the external dataset.** (A) Linear regression model between brain age and chronological age. The mean absolute error (MAE) was 0.508 and regression coefficient (*R*^2^) was 0.992. Distributions of (B) the averages and (C) standard deviations of the absolute predicted age difference (PAD) using a single volume or a single slice. During 10,000 random selections, a single volume and a single slice were randomly selected in each subject. Our method with the mode showed significantly lower MAE and standard deviation than those using a single volume or a single slice.
